# New anti-infective strategies for treatment of tularemia

**DOI:** 10.3389/fcimb.2014.00115

**Published:** 2014-08-19

**Authors:** Max Maurin

**Affiliations:** ^1^Centre National de Référence des Francisella, Institut de Biologie et Pathologie, Centre Hospitalier Universitaire de GrenobleGrenoble, France; ^2^Laboratoire Adaptation et Pathogénie des Microorganismes, Université Joseph Fourier – Grenoble 1Grenoble, France; ^3^Laboratoire Adaptation et Pathogénie des Microorganismes, CNRS/UJF, UMR 5163Grenoble, France

**Keywords:** *Francisella tularensis*, tularemia, antiinfective agents, immunomodulators, virulence

This collection of 10 articles in *Frontiers and Cellular Microbiology* aims to present the current opportunities for development of innovative therapeutic strategies for tularemia, a zoonotic disease caused by *Francisella tularensis*. This Gram-negative, facultative intracellular bacterium is a class 3 human pathogen and a CDC category A bioterrorism agent. Two *F. tularensis* subspecies are responsible for tularemia in humans, subsp. *holarctica* (type B) in the northern hemisphere and subsp. *tularensis* (type A) in North America, which themselves are split into genotypes of variable virulence. *F. tularensis* is transmitted to humans via the dermal, oral, conjunctival or respiratory routes, through direct contact with infected animals (specifically lagomorphs), ingestion of contaminated food or water, inhalation of contaminated aerosols, or arthropod bites (mainly ticks). The disease usually manifests as a regional lymphadenopathy (ulceroglandular, glandular, oculoglandular, and pharyngeal forms), or as systemic typhoid-like (typhoidal form) or pneumonic (pneumonic form) diseases. This latter form is the most feared in the context of bioterrorism. There is no vaccine for tularemia, and few antibiotics are effective in treating tularemia patients, including the aminoglycosides (streptomycin and gentamicin), the fluoroquinolones and the tetracyclines. No acquired resistance to these drugs has been reported in clinical situations, but antibiotics resistant strains could be genetically engineered for biothreat purposes. Thus, tularemia is a relevant model for development of modern anti-infective alternatives.

Because tularemia is a rare disease, clinical trials in infected humans for comparing different therapeutic strategies are not feasible. A number of *in vitro* and *in vivo* models have been developed for evaluation of new treatment alternatives. Importantly, the most relevant models are those using the highly virulent A1b genotype strains of *F. tularensis*, especially the Schu S4 strain. However, experiments using this select agent must be performed in biological safety level 3 laboratories and are submitted to drastic regulation in most countries. Thus, *F. tularensis* strains with attenuated virulence have been used for decades in research laboratories, especially the *F. novicida* Utah 112 strain. In a comparative review, Kingry and Petersen (2014) clearly demonstrate that *F. novicida* should no longer be used as a surrogate of *F. tularensis* for therapeutic efficacy testing in animal models. These two bacteria strongly differ in their natural life cycle, behavior in phagocytes, immunomodulatory properties, and pathogenicity to humans.

Potential new therapeutic strategies for tularemia include the development of new antibiotics or new ways of using existing antibiotics, the reduction of *F. tularensis* virulence and the enhancement of the host innate and/or adaptive immune response (Boisset et al., 2014). Hamblin et al. (2014) report here the high *in vivo* efficacy of a commercial formulation of liposome-encapsulated ciprofloxacin (Lipoquin®, Aradigm Corporation) in mice challenged with the Schu S4 strain. While developing a new dye uptake assay to test the activity of antibiotics against intracellular *F. tularensis*, Sutera et al. (2014) found a significant activity of linezolid (whose activity is currently restricted to Gram-positive bacteria) against intracellular *F. tularensis*. Schmitt et al. (2013) report the intracellular bactericidal activity of resazurin, a compound used for a long time to evaluate the bacterial and eukaryotic cell viability, against *F. tularensis* and *Neisseria gonorrhoeae* (gonorrhea agent). Caspar et al. (2014) present the activity of novel synthetic bis-indole derivatives against *F. tularensis*, although their cytotoxicity precluded evaluation of their activity against the intracellular form of the bacterium.

Opportunities to develop anti-infective agents inhibiting *F. tularensis* virulence or modulating the immune host response are then discussed. Jones et al. (2014) summarize the mechanisms of virulence of *F. tularensis*, including: (1) a capsule-related resistance of *F. tularensis* to opsonization and killing by the complement; (2) lack of activation of TLR4 cytokine response pathway by the lipid A of *F. tularensis* LPS leading to a poor inflammatory response of the phagocytic cells; (3) *F. tularensis* escape of phagolysosomal pathway of phagocytic cells thanks to effectors encoded by a number of genes gathered in the *Francisella* pathogenicity island (FPI); (4) alteration of several adaptive immune response pathways precluding development of an efficient host response. The intracellular multiplication of *F. tularensis* may first be altered by blocking nutrient retrieval of this bacterium within its eukaryotic host cell. As an example, Abu Kwaik (2013) report that, to increase intracellular cysteine concentration, *F. tularensis* cleaves intracellular host glutathione using a gamma-glutamyl transpeptidase, an enzyme that could represent a useful therapeutic target. *F. tularensis* is able to infect and multiply in polymorphonuclears (PMN), a rare property among pathogenic bacteria. Allen (2013) describes how *F. tularensis* turns the intrinsic properties of the PMNs to increase its pathogenicity, including PMNs longevity enhancing due to apoptosis blockade, accumulation of PMNs in infected tissues, granuloma formation and finally extensive tissue damages. Gillette et al. (2014) summarize how *F. tularensis* perturbs the host immune response by altering the intracellular signaling pathways of monocytes/macrophages. Each step of *F. tularensis-induced* dysregulation of the inflammatory process may represent a target for development of molecules capable of restoring the host immune response. Gillette et al. (2014) mention the potential clinical usefulness of available immunomodulatory compounds for treatment of severely diseased tularemia patients, including type I and type II interferons, celecoxib, imiquimod, and the granulocyte-macrophage colony-stimulating factor (GM-CSF).

In conclusion, promising opportunities exist to improve the treatment of severe forms of tularemia. However, it is probable that none of them taken separately may solve all clinical situations. The combination of one or several immunomodulatory strategies with the administration of drugs inhibiting growth and/or virulence of *F. tularensis* may represent new and interesting therapeutic alternatives, which justifies having simultaneously addressed these different aspects in the present research topic. As an editor of this research topic, I would like to greatly thank all coauthors for their valuable and interesting contributions. We wish the readers of this e-book a productive and enjoyable reading, and we hope that new ideas will emerge on this topic.

## Conflict of interest statement

The author declares that the research was conducted in the absence of any commercial or financial relationships that could be construed as a potential conflict of interest.
